# High levels of monocytic myeloid-derived suppressor cells are associated with favorable outcome in patients with pneumonia and sepsis with multi-organ failure

**DOI:** 10.1186/s40635-022-00431-0

**Published:** 2022-02-11

**Authors:** Irene T. Schrijver, Eleni Karakike, Charlotte Théroude, Pétra Baumgartner, Alexandre Harari, Evangelos J. Giamarellos-Bourboulis, Thierry Calandra, Thierry Roger

**Affiliations:** 1 Infectious Diseases Service, Department of Medicine, Lausanne University Hospital and University of Lausanne, CLED.04.407, Chemin des Boveresses 155, 1066 Epalinges, Switzerland; 2 4th Department of Internal Medicine, National and Kapodistrian University of Athens, Medical School, Athens, Greece; 3 Department of Oncology, Lausanne University Hospital and University of Lausanne, Lausanne, Switzerland

**Keywords:** Sepsis, Pneumonia, Infection, Multi-organ dysfunction, Myeloid-derived suppressor cells, Critically ill, Intensive care, APACHE II

## Abstract

**Background:**

Myeloid-derived suppressor cells (MDSCs) are immature myeloid cells with immunosuppressive functions sub-classified into monocytic and polymorphonuclear MDSCs (M-MDSCs and PMN-MDSCs). Clinical studies reported increased levels of MDSCs that were associated with poor outcome in sepsis patients. Since sepsis patients exhibit signs of inflammation and immunosuppression, MDSCs may provide benefit by dampening deleterious inflammation in some patients. To test this hypothesis, we measured MDSCs in critically ill sepsis patients with pneumonia and multi-organ dysfunctions and a high likelihood of death.

**Methods:**

This was a prospective multicenter observational cohort study performed in eight ICUs in Athens and Thessaloniki, Greece, enrolling critically ill patients with pneumonia and sepsis with multi-organ dysfunctions. A flow cytometry approach using blood collected at study inclusion in tubes containing lyophilized antibodies combined to unsupervised clustering was developed to quantify M-MDSCs and PMN-MDSCs.

**Results:**

Forty-eight patients were included, of whom 34 died within 90 days. At study inclusion, M-MDSCs and PMN-MDSCs were increased in sepsis patients when compared to healthy subjects (3.07% vs 0.96% and 22% vs 2.1% of leukocytes, respectively; *p* < 10^–4^). Increased PMN-MDSCs were associated with secondary infections (*p* = 0.024) and new sepsis episodes (*p* = 0.036). M-MDSCs were more abundant in survivors than in patients who died within 28 days (*p* = 0.028). Stratification of patients according to M-MDSC levels revealed that high levels of M-MDSC were associated with reduced 90-day mortality (high vs low M-MDSCs: 47% vs 84% mortality, *p* = 0.003, hazard ratio [HR] = 3.2, 95% CI 1.4–7.2). Combining high M-MDSC levels with low Acute Physiology and Chronic Health Evaluation (APACHE) II score improved patient stratification (M-MDSCs^high^/APACHE II^low^ vs M-MDSCs^low^/APACHE II^low^: 20% vs 80% 90-day mortality, *p* = 0.0096, HR = 7.2, 95% CI 1.6–32). In multivariate analyses high M-MDSCs remained correlated with improved survival in patients with low APACHE II score (*p* = 0.05, HR = 5.26, 95% CI 1.0–27.8).

**Conclusion:**

This is the first study to associate high levels of M-MDSCs with improved survival in sepsis patients.

**Supplementary Information:**

The online version contains supplementary material available at 10.1186/s40635-022-00431-0.

## Introduction

Sepsis is defined as a dysregulated host response to an infection resulting in life-threatening organ dysfunction [1]. The prevalence of sepsis is increasing, and recent estimations suggest that sepsis affects about 48.9 million people and is responsible of 11.0 million sepsis-related deaths per year, representing 19.7% of all deaths worldwide [2]. Sepsis survivors frequently develop functional and cognitive impairments and worsening of chronic health conditions. Almost half of patients surviving sepsis are re-hospitalized within a year [3–5].

Exuberant proinflammatory responses during the early phase of sepsis, illustrated by the so-called “cytokine storm”, are implicated in tissue damage, organ dysfunctions and early mortality. A concomitant compensatory anti-inflammatory response participating to inflammation resolution and tissue repair promotes immunosuppression that can persist for extended periods of time. Immunosuppression includes features such as apoptosis-mediated depletion of dendritic cells, T cells and B cells, decreased expression of proinflammatory cytokines, increased expression of anti-inflammatory cytokines and inhibitory checkpoint molecules, and reduced expression of antigen-presenting molecules and costimulatory molecules by immune cells [5–11]. Persistent immunosuppression favors the development of secondary infections accounting for late mortality and morbidity. Hence, immunomodulatory therapies in sepsis should target inflammation or immunosuppression depending on patient’s status. Theragnostic approaches are promising for monitoring immune status and selecting the most appropriate host-directed immunotherapy to be implemented in a personalized manner [7, 10, 12, 13].

Myeloid-derived suppressor cells (MDSCs) are immature-like myeloid cells characterized by their immunosuppressive impact on innate and adaptive immune responses [14, 15]. Generally, MDSCs are subdivided into monocytic and polymorphonuclear MDSCs (M-MDSCs and PMN-MDSCs), yet additional subtypes have been proposed among which early stage and eosinophilic MDSCs [14–17]. MDSCs are rare in blood at homeostasis but expand when inflammatory and danger signals stimulate hematopoiesis. MDSCs may also be generated through the conversion of monocytes and neutrophils into pathologically activated MDSCs [14]. MDSCs have been primarily studied in the field of cancer, a condition in which these cells are enriched in tumor environment and impair anti-tumor immunity. MDSCs can rise in the blood of cancer patients to become one of the main leukocyte subtypes [18, 19]. Clinical trials targeting MDSCs are running to counterbalance tumor-associated immunosuppression in cancer patients [20, 21].

In the field of infection and sepsis, clinical studies have shown an association between high levels of PMN-MDSCs and/or M-MDSCs in the blood and development of nosocomial infections, morbidity and/or mortality [22–31]. These observations led to the proposal that MDSCs sustain immunosuppression, and could be targeted to reverse immunosuppression in septic patients. However, clinical studies included a limited number of patients with mixed infection etiologies and medical care. Moreover, MDSCs may have different impacts depending on disease progression [7, 26, 32]. Here, we conducted a prospective clinical study in patients with sepsis due to pneumonia, multi-organ failure and high likelihood of poor outcome to characterize MDSCs in severely ill sepsis patients.

## Methods

### Study design and setting

This study was a prospective multicenter observational study performed in 8 ICUs in Athens and Thessaloniki, Greece. This was part of the INCLASS study (benefit of clarithromycin in patients with severe infections through modulation of the immune system study; registered at ClinicalTrials.gov, reference NCT03345992). The study was conducted in compliance with the declaration of Helsinki, and was approved by the central Ethics committee (52086/2017) and the National organization for Medicines-EOF (51239/01-06-2017) in Athens, Greece. Eighteen healthy volunteers serving as controls were recruited at Lausanne University Hospital. Exclusion criteria for healthy volunteers were prior diagnosis of sepsis or SARS-CoV-2 infection, acute or chronic viral hepatitis, autoimmune disease, immunodeficiency and use of immunomodulatory drugs. The study was approved by the Commission cantonale d'éthique de la recherche sur l'être humain, Canton de Vaud, Switzerland (CER-VD, Lausanne, Switzerland). Written informed consent was obtained from study participants or legal representatives prior to enrollment.

### Patients

Between December 2017 and February 2019, 48 adult patients with pneumonia were prospectively recruited from eight hospitals in Athens and Thessaloniki, Greece (Table 1). Inclusion criteria were a Sequential Organ Failure Assessment (SOFA) score ≥ 7, including respiratory failure (PiO_2_/FiO_2_ < 200), and any other organ system failure with SOFA score of ≥ 3. Most patients were on the ICU when included (*n* = 28), with a median time of 5 days (interquartile range: 2–7 days). Exclusion criteria were pregnancy, corticosteroid intake, macrolide treatment, allergy to macrolides, neutropenia (< 1000/mm^3^), HIV infection (with CD4^+^ T cells < 200/mm^3^), neoplasm or transplantation. Ethylenediamine tetraacetic acid-anticoagulated blood samples were collected at study inclusion and 5 and 10 days later. Patients were followed up for 28 days, recording all-cause mortality and incidence of secondary infections and new sepsis episodes. The definitions of ventilator-associated pneumonia (VAP), hospital-acquired pneumonia (HAP), healthcare-associated pneumonia (HCAP), secondary infections and new sepsis episodes used in the INCLASS study (available on clinicaltrials.gov) are listed in Additional file 1: Table S1. A late assessment of mortality at 90 days was performed. Mortality was coded as follows: (1) sepsis-related mortality/multi-organ failure (due to progression of the initial septic episode), (2) mortality due to secondary sepsis/infection, and (3) mortality due to other causes. Lactate and CRP levels were measured in routine laboratories of hospitals. Interleukin-6 (IL-6) and ferritin were quantified by enzyme-linked immunosorbent assay (IL-6: Invitrogen, Carlsbad, CA, lower limit of detection: 10 pg/mL; ferritin: ORGENTEC Diagnostika GmbH, Mainz, Germany, lower limit of detection: 75 ng/mL).

**Table 1:** Characteristics of healthy subjects and patients

Characteristic	Healthy controls	Survivors (90 days)	Non-survivors (90 days)	*p* value* (survivors vs non-survivors)
Number of patients	18	14	34	
Gender, male	15 (83%)	11 (79%)	24 (71%)	
Age (years)	53 [25–58]	57 [47–74]	75 [67–86]	**0.0014**
Type of infection	–			
VAP/HAP	–	11 (79%)	22 (65%)	0.35
HCAP	–	3 (21%)	12 (35%)	
Severity of illness at admission				
APACHE II score	–	16 [14–21]	23 [18–27]	**0.009**
SOFA score	–	10 [8.8–11]	10 [9–12]	0.41
Secondary infections	–	9 (64%)	17 (50%)	0.36
New sepsis episode	–	7 (50%)	16 (47%)	0.85
Charlson comorbidity index	–	4 [1–5]	6 [5–9]	**0.002**
Length of hospital stay	–	36 [24–48]	14 [8–28]	**0.003**
Length of ICU stay	–	26 [13–37]	15 [8–28]	0.16
Leukocytes (× 10^9^/L)	–	13.9 [7.5–16.0]	13.8 [10.6–20.4]	0.32
PMN-MDSCs (% of leukocytes)	2.1 [0.74–3.1]	22 [6–44]	22 [8–37]	0.96
M-MDSCs (% of leukocytes)	0.96 [0.46–1.5]	4.6 [2.6–6.5]	2.9 [1.8–4.1]	0.052
Lactate (mmol/L)	–	1.4 [0.85–2.2]	2.8 [1.6–2.8]	**0.02**
CRP (mg/L)	–	130 [40–174]	144 [78–184]	0.63
IL-6 (pg/mL)	–	29 [19–52]	31 [17–73]	0.73
Ferritin (ng/mL)	–	501 [349–675]	748 [437–1478]	0.14

*p* values < 0.05 are highlighted in bold

Data are medians [IQR] or *n* (%). Severity scores, leukocyte counts, MDSC levels and lactate levels were measured at study inclusion

*VAP* ventilator-associated pneumonia, *HAP* hospital-acquired pneumonia, *HCAP* healthcare-associated pneumonia, *PMN-MDSCs* polymorphonuclear-MDSCs, *M-MDSCs* monocytic myeloid-derived suppressor cells

^*^p values comparing survivors and non-survivors at 90 days

### Flow cytometry analysis

We established a procedure to limit variability resulting from sample handling/labeling and analysis. To that end, we used a targeted flow cytometry approach using blood samples collected in tubes containing a mixture of lyophilized fluorescently labeled antibodies targeting MDCSs developed in collaboration with DURAClone (Beckman Coulter, Brea, CA). These tubes contained antibodies (clone/fluorochrome) directed against human CD3 (UCHT1/APC-AF700), CD11b (Bear1/PE-Cy7), CD14 (RMO52/APC-AF750), CD15 (80H5/Pacific Blue), CD16 (3G8/ECD), CD19 (J3-119/APC-AF700), CD33 (D3HL60.251/APC), CD45 (J33/Krome Orange), CD56 (NKH-1/APC-AF700), CD124 (G077F6/PE) and human leukocyte antigen (HLA)-DR (Immu-357/FITC). CD3, CD19, CD56 labeled with the same fluorochrome were used to filter lineage-positive leukocytes. One hundred microliter of blood were added to the tubes (all from the same batch) and gently pipetted up and down 10 times. After 20 min, 900 μL of 1 × BD FACS™ lysing solution (BD Biosciences, San Jose, CA) were added. Samples were vortexed and frozen at − 80 °C until all samples were acquired. Samples were thawed, washed with cell stain medium (PBS containing 0.5% BSA and 0.02% sodium azide) and acquired in a single day using an Attune NxT Flow Cytometer (Thermo Fisher Scientific, Waltham, MA, USA). Debris, doublets and CD45-negative cells were excluded from analysis by manual gating using FlowJo™ (v10.6.2, Ashland, OR) (Additional file 2: Fig. S1). We then applied FlowSOM for unsupervised clustering using the biexponential transformed and normalized expression levels of cell surface markers CD3/CD19/CD56 (lineage), CD11b, CD14, CD15, CD16, CD33, CD45, CD124 and HLA-DR and relative side scatter area (SSC-A). Metaclusters were set on 30 populations, merged into 8 populations based on biological knowledge and marker expression, as represented in tSNE and heatmap plots. M-MDSCs corresponded to CD11b^+^ CD14^+^ CD15^−/low^ CD16^−^ CD33^+^ HLA-DR^−/low^ cells, while PMN-MDSCs corresponded to CD11b^+^ CD14^−^ CD15^+^ CD16^+^ CD33^−^ HLA-DR^−^ cells.

### Statistical analysis

Baseline patient characteristics were compared using Chi-square exact test, Mann–Whitney *U* test, and Kruskal–Wallis test as appropriate. The comparison between cell populations and clinical data were evaluated using the Mann–Whitney *U* test and correlation studies performed using Spearman’s rank-order correlation. M-MDSCs ≤ 4.3% and > 4.3% of leukocytes were considered as low and high percentages, respectively. The cutoff value was based on highest tertile of % M-MDSCs in sepsis patients. APACHE II scores ≤ 20 and > 20 (cutoff values based on median) were considered as low and high. Statistical differences between survival and event curves were assessed with the log-rank test. The contribution of MDSCs to mortality was analyzed using the Cox proportional hazard model. Statistics and figure design were performed using R v.3.6.0 (R-Foundation for Statistical Computing, Vienna, Austria). *p* values < 0.05 were considered to be statistically significant.

## Results

### Clinical data

We included 48 patients with sepsis due to pneumonia (33 [69%] with ventilator-associated/hospital-acquired pneumonia and 15 [31%] with healthcare-associated pneumonia). Fourteen patients (29.2%) survived, 23 patients (47.9%) died within 28 days and 34 patients (70.8%) died within 90 days (Table 1). The median age of 90-day survivors was significantly lower than the median age of 90-day non-survivors (median and interquartile range [IQR]): 57 [47–74] vs 75 [67–86] years; *p* = 0.0014). At study inclusion, 90-day survivors and non-survivors had similar SOFA scores, leukocyte counts, and C-reactive protein (CRP), IL-6 and ferritin levels. Survivors had lower APACHE II scores (16 [14–21] vs 23 [18–27]; *p* = 0.009), Charlson comorbidity indexes (4 [1–5] vs 6 [5–9]; *p* = 0.002) and lactate levels (1.4 [0.85–2.2] vs 2.8 [1.6–2.8] mmol/L; *p* = 0.02). Survivors had longer length of hospital stays (36 [24–48] vs 14 [8–28] days; *p* = 0.003) (Table 1).

### Differential expression of M-MDSCs and PMN-MDSCs in sepsis patients

**Fig. 1:**
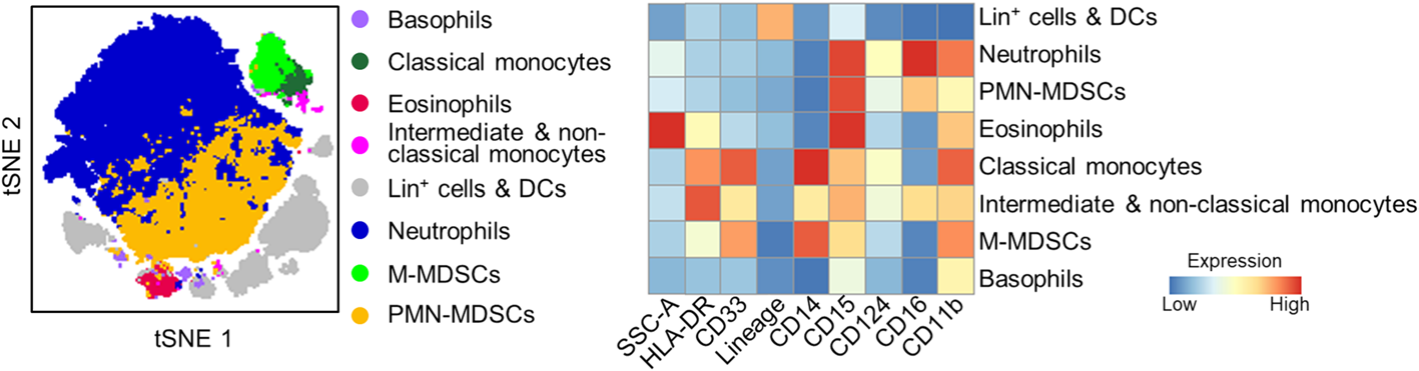
Identification of MDSCs by flow cytometry and unsupervised clustering. Blood was collected in tubes containing lyophilized fluorescently labeled antibodies targeting MDCSs and analyzed as described in “Methods”. t-SNE plots of leukocyte populations (left) and relative side scatter area (SSC-A) and expression levels of surface markers (right). Lin+: lineage (i.e., CD3, CD19 or CD56) positive; DCs: dendritic cells

We used a targeted flow cytometry approach combined to unsupervised automatic clustering to identify M-MDSCs and PMN-MDSCs in whole blood (see “Methods” and Fig. 1). M-MDSCs and PMN-MDSCs represented 0.96% [0.46–1.5] and 2.1% [0.7–3.1] of leukocytes in healthy individuals (*n* = 18), respectively. Considering all sepsis patients analyzed at study inclusion, M-MDSCs and PMN-MDSCs represented 3.1% [2.04–4.85] and 22% [7.9–43.0] of leukocytes (*p* < 10^–4^ vs healthy individuals) (Additional file 1: Table S2). The percentages and absolute counts of M-MDSCs and PMN-MDSCs remained elevated and were not statistically significantly altered over a 10-day follow-up period (Additional file 2: Fig. S2). Therefore, subsequent analyses were performed using the levels of M-MDSCs and PMN-MDSCs measured at study inclusion.

**Fig. 2:**
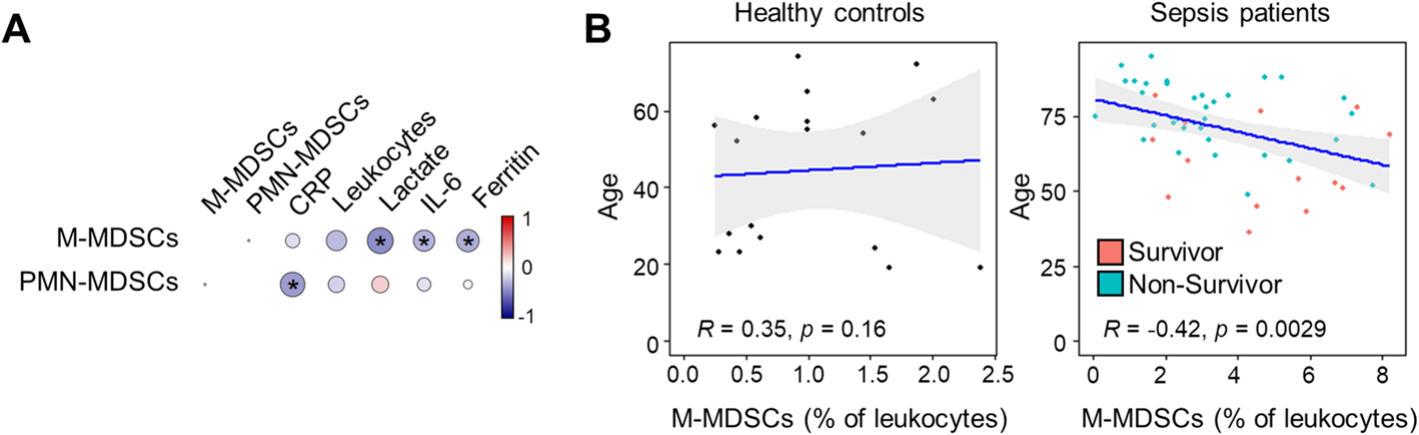
**A** Correlation plot matrix of M-MDSCs, PMN-MDSCs, CRP, leukocytes, lactate, IL-6 and ferritin levels. *M-MDSCs inversely correlated with lactate (*ρ* = − 0.43, *p* = 0.002), IL-6, (*ρ* = − 0.29, *p* = 0.045), and ferritin (*ρ* = − 0.32, *p* = 0.028), and PMN-MDSCs inversely correlated with CRP (*ρ* = − 0.39, *p* = 0.047). **B** Scatterplots of M-MDSCs (% of leukocytes) and age in healthy controls (left) and sepsis patients (right)

The percentages of M-MDSCs at study inclusion showed a trend towards lower levels in 90-day non-survivors when compared to survivors (2.9% [1.8–4.1] vs 4.6% [2.6–6.5]; *p* = 0.052) (Table 1). The percentages of M-MDSCs did not correlate with the percentages of PMN-MDSCs (Spearman’s correlation coefficient [*ρ*] = − 0.003, *p* = 0.98) (Fig. 2A). M-MDSCs inversely correlated with lactate levels (*ρ* = − 0.43, *p* = 0.002), IL-6 levels, (*ρ* = − 0.29, *p* = 0.045), and ferritin levels (*ρ* = − 0.32, *p* = 0.028) (Fig. 2A), while PMN-MDSCs inversely correlated with CRP levels (*ρ* = − 0.39, *p* = 0.047). M-MDSCs but not PMN-MDSCs inversely correlated with the age of sepsis patients, while no such correlation was observed in the group of healthy controls for both M-MDSCs and PMN-MDSCs (Fig. 2B and Additional file 2: Fig. S3).

**Fig. 3:**
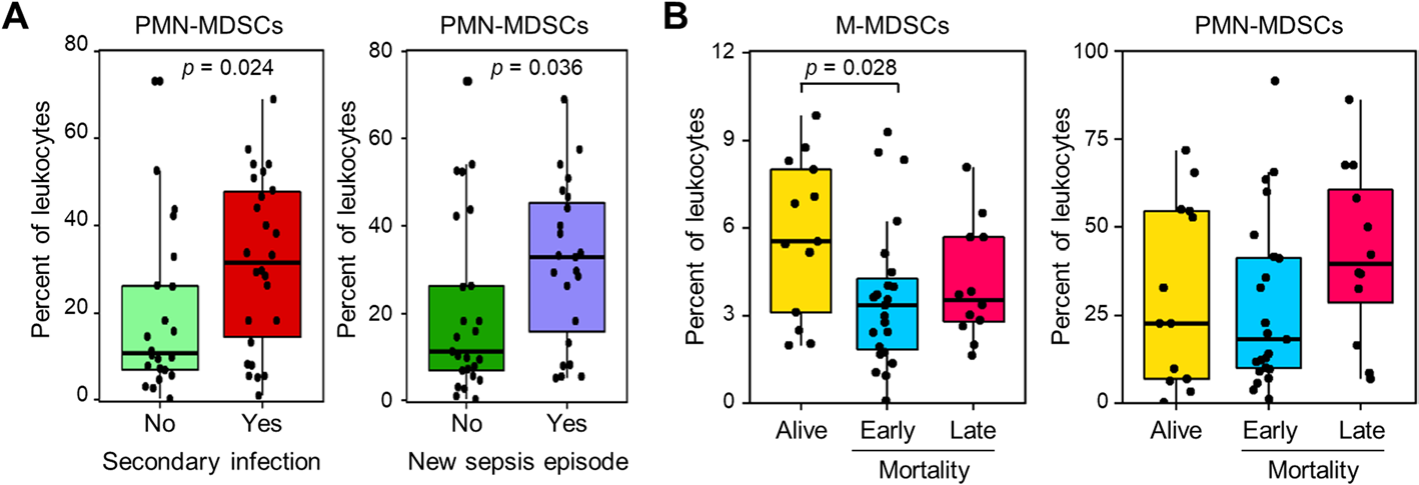
**A** PMN-MDSCs in relation with secondary infection and new sepsis episode. **B** M-MDSCs and PMN-MDCs in survivors (*n* = 14), early deaths (≤ 28 days, *n* = 23) and late deaths (> 28 days, *n* = 12). Boxplots show median, upper and lower quartiles. Whiskers show 5 to 95 percentiles. Each dot represents an individual sample

Twenty-six (66.7%) patients developed a secondary infection, among which 23 (47%) were associated with a new sepsis episode (see definitions in Additional file 1: Table S1). Patients who developed or not a secondary infection were similar in age and gender, had similar APACHE II and SOFA scores at admission, and comparable 90-day mortality rates. However, patients who developed a secondary infection stayed 3.6- to 4.1-fold longer in hospital (33 [27–43] vs 8 [5–12] days; *p* < 0.0001) and ICUs (29 [20–36] vs 8 [5–10] days; *p* < 0.0001) and had lower CRP levels (75 [27–144] vs 164 [130–194] mg/L; *p* = 0.003) than patients who did not develop a secondary infection (Table 2). Moreover, patients who developed a secondary infection presented higher levels of PMN-MDSCs than patients that did not develop a secondary infection (31% [13–46] vs 11% [7–26]; *p* = 0.03) and new sepsis episode (33% [14–45] vs 11% [7–26]; *p* = 0.04) (Fig. 3A). Besides, patients whose mortality was related to secondary sepsis/infection expressed 3.7-fold higher levels of PMN-MDSCs than patients whose mortality was related to the primary sepsis event (36% [26–49] vs 9.8% [7.3–14.5] of leukocytes, *p* = 0.0021) (Additional file 2: Fig. S4). No difference in M-MDSCs was observed for all these parameters. PMN-MDSCs and M-MDSCs were similarly represented in patients with documented Gram-negative (*n* = 23) and Gram-positive (*n* = 8) infections.

**Table 2:** Characteristics of patients grouped according to the occurrence of secondary infection and M-MDSC level

Characteristic	No secondary infection	Developed a secondary infection	*p *value	M-MDSCs ≤ 4.3%	M-MDSCs > 4.3%	*p* value
Number	22	26		31	17	
Gender, male	14 (64%)	21 (81%)	0.18	22 (71%)	13 (76%)	0.17
Age (year)	74 [63–85]	73 [62–80]	0.46	74 [69–82]	62 [52–77]	0.6
Type of infection						
HAP/VAP	12 (55%)	19 (73%)	0.18	18 (68%)	13 (76%)	0.2
HCAP	10 (45%)	7 (27%)		13 (42%)	4 (24%)	
Severity of illness at admission						
APACHE II score	20 [17–25]	20 [16–26]	0.99	21 [17–27]	20 [15–23]	0.29
SOFA score	11 [9–12]	10 [9–12]	0.60	10 [9–12]	11 [10–12]	0.61
Mortality day 90	17 (77%)	17 (65%)	0.37	26 (84%)	8 (47%)	**0.007**
New sepsis episode	–	–		16 (52%)	7 (41%)	0.48
Secondary infection	–	–		17 (55%)	9 (53%)	0.90
Length of hospital stay	8 [5–12]	33 [27–43]	**< 0.0001**	16 [8–28]	33 [12–44]	**0.04**
Length of ICU stay	8 [5–10]	29 [20–36]	< 0.0001	16 [8–26]	27 [10–37]	0.14
Leukocytes (× 10^9^/L)	14.6 [11.6–19.6]	13.5 [10.0–17.9]	0.59	15.5 [12.1–20.4]	10.9 [7.2–16.9]	**0.04**
PMN-MDSCs (% of leukocytes)	11 [7–26]	32 [14–48]	**0.02**	26 [9–39]	18 [6–44]	0.86
M-MDSCs (% of leukocytes)	3.04 (1.72–6.48)	3.07 (2.12, 4.60)	0.83	–	–	
Lactate (mmol/L)	2.10 [1.70–2.80]	1.70 [1.20–2.30]	0.28	2.18 [1.70–2.80]	1.20 [0.90–2.10]	**0.01**
CRP (mg/L)	164 [130–194]	75 [27–144]	**0.003**	146 [81–218]	126 [40–167]	0.23
IL-6 (pg/mL)	43 [22–112]	24 [16–50]	0.07	31 [16–72]	29 [20–49]	0.89
Ferritin (ng/mL)	850 [410–1533]	530 [391–981]	0.40	747 [476–1524]	423 [266–808]	**0.03**

*p* values < 0.05 are highlighted in bold

Data are medians [IQR] or *n* (%). Leukocytes, the MDSC-populations and lactate were assessed at study inclusion. Cut-off values of M-MDSCs is expressed in % of leukocytes. *p* values < 0.05 are highlighted in bold

*VAP* ventilator-associated pneumonia, *HAP* hospital-acquired pneumonia, *HCAP* healthcare-associated pneumonia, *PMN-MDSCs* polymorphonuclear-MDSCs, *M-MDSCs* monocytic myeloid-derived suppressor cells

Survivors and early and late deaths (i.e., ≤ 28 and > 28 days) expressed similar levels of PMN-MDSCs (Fig. 3B). In contrast, survivors expressed 1.64-fold more M-MDSCs than early deaths (4.6% [2.6–6.7] vs 2.8% [1.5–3.6], *p* = 0.028) and, albeit not significant, 1.55-fold more M-MDSCs than late deaths (3.0% [2.3–4.7], *p* = 0.19) (Fig. 3B). Subsequently, we stratified patients according to the expression of M-MDSCs (low and high levels: ≤ 4.3% and > 4.3%) and the APACHE II score (low and high: ≤ 20 and > 20) (see “Methods”).

**Fig. 4:**
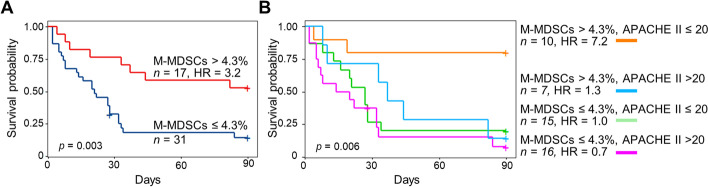
Kaplan–Meier 90-day survival curves based on low and high levels of M-MDSCs (≤ 4.3% and > 4.3% of leukocytes) (**A**) and on the combination of low and high levels of MDSCs and low and high APACHE II scores (≤ 20 and > 20) (**B**). Statistical differences were assessed using the log-rank test

Ninety-day mortality was decreased in patients with high levels of M-MDSCs (high vs low MDSCs: 47% vs 84% mortality, *p* = 0.007, hazard ratio [HR] = 3.2, 95% confidence interval [95% CI] 1.4–7.2) (Table 2 and Fig. 4A), while hospital stay was increased (high vs low MDSCs: 33 [12–44] vs 16 [8–28] days; *p* = 0.04) (Table 2). Patients with high levels of M-MDSCs showed reduced leukocytes counts, lactate levels and ferritin levels at admission (*p* = 0.04, 0.01 and 0.03, respectively) (Table 2).

Combining M-MDSCs (low and high levels) and APACHE II score (low and high) in analyses increased patient stratification. The 90-day mortality rate was 20% in patients with high M-MDSCs and low APACHE II score, while it was 71–88% in the three other groups (overall comparison: *p* = 0.0062; M-MDSCs^high^/APACHE II^low^ vs M-MDSCs^low^/APACHE II^low^: 20% vs 80%, *p* = 0.0096, HR = 7.2, 95% CI 1.6–32) (Fig. 4B). In multivariate analyses including baseline factors associated with mortality (age, APACHE II score, Charlson comorbidity index), high M-MDSCs remained associated with improved survival in patients with low APACHE II score (*p* = 0.05, HR = 5.26, 95% CI 1.0–27.8) (Table 3).

**Table 3:** Multivariate analyses of variables associated with survival in sepsis patients

Variable	*p* value	HR	95% CI HR
All patients			
Age	0.08	1.04	1.00–1.08
Apache II	0.92	1.00	0.93–1.08
Charlson comorbidity index	0.27	1.18	0.92–1.36
M-MDSCs > 4.3%	0.10	2.06	0.87–4.92
Patients with APACHE II score ≤ 20			
Age	0.41	1.03	0.95–1.12
Apache II	0.30	1.15	0.88–1.50
Charlson comorbidity index	0.21	1.27	0.87–1.87
M-MDSCs > 4.3%	**0.05**	5.26	1.00–27.8

*p* value < 0.05 is highlighted in bold

## Discussion

To our knowledge, this is the first study reporting that high expression levels of M-MDSCs are associated with improved outcome of sepsis patients with pneumonia.

M-MDSCs and PMN-MDSCs remained stably elevated during 10 days of follow-up. These data corroborate the persistence of MDSCs for 14 to 28 days in sepsis and ICU surgical patients, and of M-MDSCs and PMN-MDSCs for 8 days in sepsis patients [23, 27–29]. M-MDSCs and PMN-MDSCs were similarly expressed in patients with Gram-negative and Gram-positive infections, while few studies reported the preferential expansion of M-MDSCs and PMN-MDSCs in Gram-negative infections and Gram-positive infections, respectively [22, 25]. Thus, while chronical elevation of MDSCs may be an attractive biomarker for sepsis [9, 26, 32], additional studies will be required to outline whether the expansion of specific subpopulations of MDSCs in sepsis results from different kinds of infections. We did not detect a correlation between the levels of M-MDSCs and PMN-MDSCs. This might suggest that, under pathological conditions, these populations result from different hematopoietic drivers [33].

Experimental investigations and all clinical studies to date suggested that MDSCs are detrimental during sepsis [7, 22–31]. High levels of MDSCs at admission correlated with early mortality of surgical septic shock patients [23], and high levels of M-MDSCs on days 6–8 correlated with mortality and secondary infections in septic shock patients [27]. Persistent expansion of MDSCs might be implicated in the establishment of persistent inflammation, immunosuppression and catabolism syndrome (PICS) observed in a subset of chronic critically ill patients (CCI) who experienced sepsis [34]. In our cohort, high levels of PMN-MDSCs were not associated with mortality, but were associated with the occurrence of secondary infections and new sepsis episodes. It is possible that either an association with mortality was missed because of sample size, or that the absence of association was genuine. In line with the second option, a recent study failed to detect an association between mortality and the expansion of PMN-MDSCs in blood sampled from sepsis patients at ICU admission and 3 days later [35].

Somehow astonishing, no human study ever reported a positive role of MDSCs during sepsis. Yet, previous studies included patients with diverse infection etiologies and medical care (ED, medical/surgical ICUs), while we enrolled ICU patients with severe ventilator, hospital, and healthcare-associated pneumosepsis. We cannot exclude that a beneficial role of M-MDSCs is restricted to this kind of patients. In the same line of idea, patients with sepsis caused by CAP displayed a specific blood gene expression signature on ICU admission [36]. Moreover, sepsis patients with pneumonia were differentially distributed into Mars1–4 endotypes than sepsis patients with peritonitis [37]. Therefore, the site of infection and/or surgical intervention may affect blood gene expression profile, reflecting different cellular fates.

One could imagine that MDSCs play a dual role during sepsis. Although hypothetical, this supposition is founded on several facts. First, MDSCs are phagocytic cells, which can help fighting infections through ingestion and killing of microorganisms. Second, MDSCs can dampen systemic or local inflammation induced by molecular patterns of pathogen or endogenous origin, the latter being released upon stress or during tissue injury. Third, MDSCs harvested from septic mice protected recipient mice from acute lethal infections including cecal ligation and puncture-induced sepsis and *Pseudomonas* pneumonia [38–41]. Remarkably, we observed that high levels of M-MDSCs were associated with reduced 90-day mortality in sepsis patients with multi-organ failure and high likelihood of mortality. Increased M-MDSCs might drive beneficial effects through dampening inflammation-induced organ dysfunction in severely ill sepsis patients. Of importance, high levels of M-MDSCs were not beneficial in patients with a high APACHE II score at study enrollment. Albeit speculative, a possible explanation is that these patients were on a trajectory more or less invariably conducting to death, and that the influence of M-MDSCs in those conditions was negligible.

As observed in other conditions, PMN-MDSCs were more abundant than M-MDSCs in sepsis patients (and healthy controls). It has been shown that M-MDSCs are more potent immunosuppressive cells than PMN-MDSCs on a per cell basis [26]. This may explain, at least in part, how a minor subpopulation of MDSCs may have a significant impact. Supporting the concept of MDSCs as inflammatory brakes, M-MDSCs negatively correlated with lactate, IL-6 and ferritin levels, and PMN-MDSCs with CRP levels. M-MDSCs and PMN-MDSCs mediate immunosuppressive functions through different mechanisms involving, for example the expression of IL-10, transforming growth factor β, nitric oxide and programmed death-ligand 1 (PDL1) by M-MDSCs and the expression of arginase 1 by PMN-MDSCs. PMN-MDSCs may also preferentially use reactive oxygen species, peroxynitrite and possibly prostaglandin E_2_ to drive immunosuppression [14, 42–46].

Several factors may explain discrepancies reported in the literature such as the causative agent and site of infection, the inflammatory status which impacts on myelopoiesis and the generation of MDSCs, the timing of blood sampling and downstream treatment, or the immunophenotyping of MDSCs [48]. Indeed, both mouse and human studies revealed that MDSCs evolve after sepsis onset, acquiring superior suppressive functions over time [29, 47]. A pitfall of (early) studies on MDSCs is the lack of harmonization of sample handling, and eventually the lack of discrimination of MDSC subpopulations. For instance, MDSCs are better detected in whole blood than in PBMCs [49]. PMN-MDSCs but not M-MDSCs are sensitive to freezing/thawing of PBMCs, while M-MDSCs are more sensitive than PMN-MDSCs to delayed blood processing [50, 51]. Even though using a consensus protocol, the multicenter Mye-EUNITER MDSC Monitoring Initiative reported important center-related differences in PMN-MDSCs detection in the blood healthy donors [52]. To minimize analytical variations, we labeled whole blood immediately after drawing using DURAClone tubes, and used unsupervised clustering strategies to analyze flow cytometry data. However, as often inevitable in multicenter studies, samples were cryopreserved before analysis. Finally, we acknowledge that there is still no definite perfect phenotyping protocol of MDSCs. Unbiased transcriptomics and unsupervised flow and mass cytometry might help identifying new markers of MDSCs, such as LDL receptor 1 (LOX-1) expressed by PMN-MDSCs [53–55].

Our study has several limitations including the characterization of MDSCs by phenotypic and not functional analyses, and the absence of immunological correlates. Yet, several studies reported the immunosuppressive function of MDSCs based on their phenotype [22, 25, 56]. The sample size may have affected the detection of associations between MDSCs and sepsis parameters. We focused on patients with sepsis due to pneumonia, while the role of M-MDSCs may vary in different disease processes. Patients were aged, and our observations may not be verified in a younger population. Aging is a condition that might influence MDSCs [57]. However, contrary to expectations, we detected a negative correlation between age and M-MDSCs in sepsis patients. As recently argued, it might be difficult to differentiate an increase of MDSCs to due aging (as a consequence of inflammageing) from that due to disease-mediated expansion [58]. Finally, the risk of mortality in our study population was high (71% of patients died within 90 days). However, poor outcome likely represented a favorable condition to detect a positive role of MDSCs in sepsis.

## Conclusions

This represents the first report of an association between high levels of M-MDSCs and improved outcome of patients with pneumosepsis. We believe that these observations should provide impetus for additional studies to appreciate the role of MDSCs in patients with severe sepsis and multi-organ failure, and for deciphering the mechanisms regulating the expansion and the activation of MDSCs in bacterial sepsis. Such investigations will be required to assess whether MDSCs are prognostic and/or theragnostic biomarkers in sepsis.

## Supplementary Information

**Additional file 1: Table S1.** Definitions used in the study. **Table S2.** MDSCs in healthy subjects and sepsis patients.

**Additional file 2: Fig. S1. **Gating strategy to exclude doublets and non-hematopoietic (CD45-) cells. **Fig. S2. **M-MDSCs and PMN-MDSCs expressed in % of leukocytes and absolute counts in healthy controls, and in sepsis survivors and non-survivors analyzed at days 1 (study inclusion), 5 and 10. Boxplots show median, upper and lower quartiles. Whiskers show 5 to 95 percentiles. Each dot represents an individual sample. No significant differences were detected in longitudinal analyses. **Fig. S3. **Scatterplots of PMN-MDSCs and age in healthy controls (left) and sepsis patients (right). **Fig. S4. **MDSCs (in % of leukocytes) in relation with the cause of 90-day mortality (primary sepsis related mortality *n* = 13; due to secondary infection/sepsis *n* = 16; other causes *n* = 6). Boxplots show median, upper and lower quartiles. Whiskers show 5 to 95 percentiles. Each dot represents an individual sample.

## Data Availability

The data that support the findings of this study are available from the corresponding author on reasonable request. Restrictions apply to the availability of data associated with the INCLASS study, which is not finalized (contact Prof E.J. Giamarellos-Bourboulis).

## Abbreviations

APACHE II: Acute Physiology and Chronic Health Evaluation II
CRP: C-reactive protein
HAP: Hospital-acquired pneumonia
HCAP: Healthcare-associated pneumonia
HR: Hazard ratio
IL-6: Interleukin-6
IQR: Interquartile range
MDSCs: Myeloid-derived suppressor cells
PMN-MDSCs: Polymorphonuclear-MDSCs
M-MDSCs: Monocytic myeloid-derived suppressor cells
SOFA: Sequential Organ Failure Assessment
VAP: Ventilator-associated pneumonia
95% CI: 95% Confidence interval
