# Supplementary material for: High levels of monocytic myeloid-derived suppressor cells are associated with favorable outcome in patients with pneumonia and sepsis with multi-organ failure
Source: Intensive Care Med Exp. 2022 Feb 11;10:5. doi: 10.1186/s40635-022-00431-0 (PMC8831012; doi:10.1186/s40635-022-00431-0)
Supplement: Supplementary file 1 — Additional file 1: Table S1. Definitions used in the study. Table S2. MDSCs in healthy subjects and sepsis patients. [file 40635_2022_431_MOESM1_ESM.docx]

**Additional file 1: Table S1. Definitions used in the study**

| **Hospital-acquired pneumonia (HAP)** (1) is defined by the presence of a new or progressive radiographic lung infiltrate in a non-intubated patient hospitalized for more than 48 hours who presents with at least two of the following clinical features: |
| --- |
| - Core temperature equal or greater than 38°C |
| - Total white blood cell count more than 12,000/mm3 |
| - Rales or bronchial breath sounds on physical examination |
| - Purulent sputum |
| - More than 20 breaths/minute |
| - Serum procalcitonin more than 0.25 ng/ml |
| - Gram stain of tracheobronchial secretions or bronchoalveolar lavage fluid indicating the predominance of Gram-negative bacilli |
| **Health-care associated pneumonia (HCAP)** is defined by the presence of a new or progressive radiographic lung infiltrate in a non-intubated patient who has at least one of the following risk factors for HCAP (1): |
| - Hospitalization the last 90 days |
| - Residency in a long-term care facility |
| - Under regular hemodialysis |
| AND who presents with at least two of the following clinical features: |
| - Core temperature equal or greater than 38°C |
| - Total white blood cell count more than 12,000/mm3 |
| - Rales or bronchial breath sounds on physical examination |
| - Purulent sputum |
| - More than 20 breaths/minute |
| - Serum procalcitonin more than 0.25 ng/ml |
| - Gram stain of tracheobronchial secretions or bronchoalveolar lavage fluid indicating the predominance of Gram-negative bacilli |
| **Ventilator-associated pneumonia (VAP)** is defined by the presence of a new or progressive radiographic lung infiltrate in a patient who is under mechanical ventilation for at least 48 hours AND who presents with at least two of the following clinical features (1): |
| - Core temperature equal or greater than 38°C |
| - Total white blood cell count more than 12,000/mm3 |
| - Purulent tracheobronchial secretions |
| - Serum procalcitonin more than 0.25 ng/ml |
| - Gram stain of tracheobronchial secretions or bronchoalveolar lavage fluid indicating the predominance of Gram-negative bacilli |
| **New sepsis episode** is noted in any patient who experiences at least 25% decrease of day 1 SOFA score on day 7 and who has further increase of day 7 total SOFA by at least 2 points, consequent to infection. |
| **Secondary infections** were defined by conventional criteria (2,3). Only clinically relevant, systematic infections (HAP, VAP, intra-abdominal infection, primary bacteremia, catheter-related bloodstream infection, acute bacterial skin and soft structures infection, acute pyelonephritis, catheter-associated urinary tract infection with systemic symptoms, invasive mycosis), were considered as secondary infections. |

1. Kalil AC, Metersky ML, Klompas M, Muscedere J, Sweeney DA, Palmer LB, et al. Management of adults with hospital-acquired and ventilator-associated pneumonia: 2016 clinical practice guidelines by the Infectious Diseases Society of America and the American Thoracic Society. **Clin Infect Dis 2016**; 63: e61-e111.
2. Calandra T, Cohen J; International Sepsis Forum Definition of Infection in the ICU Consensus Conference. The international sepsis forum consensus conference on definitions of infection in the intensive care unit. **Crit Care Med. 2005** Jul;33(7):1538-48.
3. Bennet JE, Dolin R, Blaser MJ. Mandell, Douglas and Bennett's Principles and practice of infectious diseases. Philadelphia, PA :Elsevier/Saunders; 2015.

# Additional file 1: Table S2. MDSCs in healthy subjects and sepsis patients

| **Characteristic** | **Control**  **(*n* = 18)** | **Sepsis patients**  **(*n* = 48)** | ***p value*** |
| --- | --- | --- | --- |
| Gender, male | 15 (83%) | 35 (73%) | 0.379 |
| Age (years) | 53 [25-58] | 73 [62-82] | **<0.0001** |
| M-MDSCs (x 10^9^ cells/L) | 0.03 [0.01-0.05] | 0.20 [0.08-0.33] | **<0.0001** |
| M-MDSCs (% of leukocytes) | 0.96 [0.46-1.5] | 3.07 [2.04-4.85] | **<0.0001** |
| PMN-MDSCs (x 10^9^ cells/L) | 0.07 [0.03-0.13] | 1.00 [0.20-2.43] | **<0.0001** |
| PMN-MDSCs (% of leukocytes) | 2.1 [0.74-3.1] | 22.0 [7.9-43.0] | **<0.0001** |

Data are medians [IQR] or n (%). Leukocytes, the MDSC-populations and lactate were assessed at study inclusion. VAP: ventilator associated pneumonia, HAP: hospital acquired pneumonia, HCAP: healthcare-associated pneumonia, PMN-MDSCs: polymorphonuclear-MDSCs, M-MDSCs: monocytic-myeloid-derived suppressor cells. Cut-off values of M-MDSCs is expressed in % of leukocytes.
