# Supplementary material for: High levels of monocytic myeloid-derived suppressor cells are associated with favorable outcome in patients with pneumonia and sepsis with multi-organ failure
Source: Intensive Care Med Exp. 2022 Feb 11;10:5. doi: 10.1186/s40635-022-00431-0 (PMC8831012; doi:10.1186/s40635-022-00431-0)
Supplement: Supplementary file 2 — Additional file 2: Fig. S1. Gating strategy to exclude doublets and non-hematopoietic (CD45-) cells. Fig. S2. M-MDSCs and PMN-MDSCs expressed in % of leukocytes and absolute counts in healthy controls, and in sepsis survivors and non-survivors analyzed at days 1 (study inclusion), 5 and 10. Boxplots show median, upper and lower quartiles. Whiskers show 5 to 95 percentiles. Each dot represents an individual sample. No significant differences were detected in longitudinal analyses. Fig. S3. Scatterplots of PMN-MDSCs and age in healthy controls (left) and sepsis patients (right). Fig. S4. MDSCs (in % of leukocytes) in relation with the cause of 90-day mortality (primary sepsis related mortality n = 13; due to secondary infection/sepsis n = 16; other causes n = 6). Boxplots show median, upper and lower quartiles. Whiskers show 5 to 95 percentiles. Each dot represents an individual sample. [file 40635_2022_431_MOESM2_ESM.pdf]

Additional file 2: supplementary figures

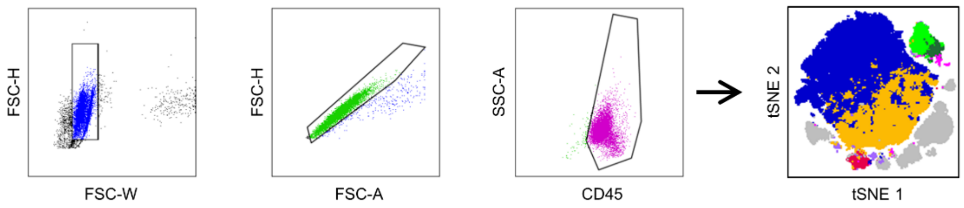

Fig. S1. Gating strategy to exclude doublets and non-hematopoietic ($CD45^-$) cells.

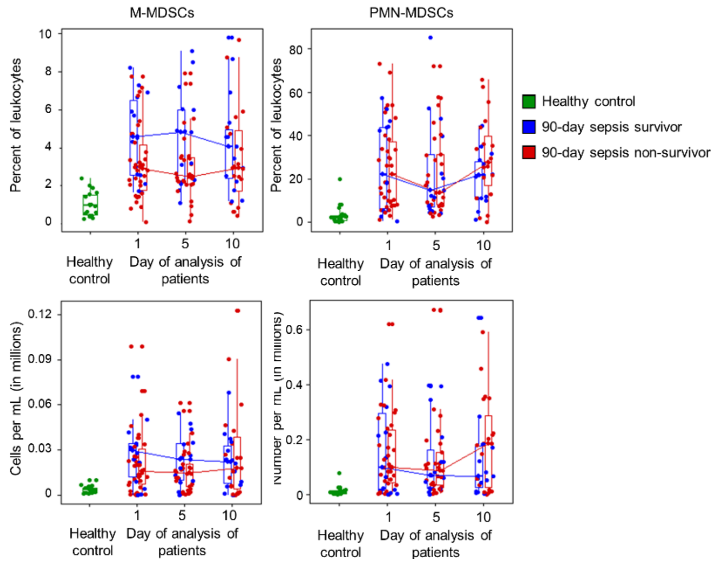

Fig. S2 M-MDSCs and PMN-MDSCs expressed in % of leukocytes and absolute counts in healthy controls, and in sepsis survivors and non-survivors analyzed at days 1 (study inclusion), 5 and 10. Boxplots show median, upper and lower quartiles. Whiskers show 5 to 95 percentiles. Each dot represents an individual sample. No significant differences were detected in longitudinal analyses.

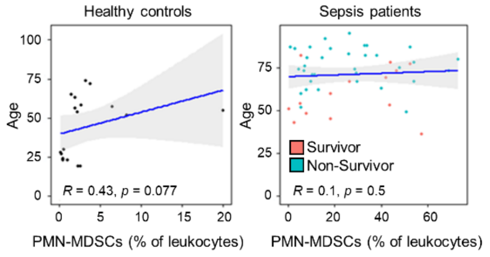

Fig. S3 Scatterplots of PMN-MDSCs and age in healthy controls (left) and sepsis patients (right).

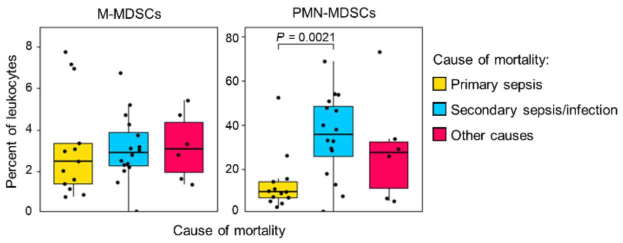

Fig. S4. MDSCs (in % of leukocytes) in relation with the cause of 90-day mortality (primary sepsis related mortality *n* = 13; due to secondary infection/sepsis *n* = 16; other causes *n* = 6). Boxplots show median, upper and lower quartiles. Whiskers show 5 to 95 percentiles. Each dot represents an individual sample.
